# Choice versus no choice: Practical considerations for increasing choices

**DOI:** 10.1002/jaba.2920

**Published:** 2024-11-12

**Authors:** Ji Young Kim, Cody Morris, Megan E. Ellsworth, Xiaoyuan Liu, Nicole F. Seacord

**Affiliations:** ^1^ School of Behavioral Sciences and Education Pennsylvania State University–Harrisburg Middletown PA USA; ^2^ Department of Psychology Salve Regina University Newport RI USA; ^3^ Health Studies & Applied Educational Psychology, Teachers College Columbia University New York NY USA

**Keywords:** choice, concurrent chains, concurrent operants, no choice, preference assessment

## Abstract

Choice involves engaging in a selection response when multiple options are concurrently available. Choices can be incorporated into many components of behavior‐analytic treatment such as providing clients with a choice between multiple items, activities, or tasks. We reviewed the main characteristics of 38 behavior‐analytic articles that compared choice and no‐choice conditions. We coded the experimental arrangements of choice and no‐choice conditions and analyzed potential factors affecting preferences for choice and no choice. The findings suggest that the sizing of alternatives from which to choose, the timing of choice opportunities, and the timing of the delivery of the chosen option varied across the studies. Furthermore, preferences for choice shifted with differential reinforcement history and response effort manipulations of choice or no choice. The findings suggest that individual variables should be considered when providing choices, but more research is needed.

In behavior analysis, choice is often conceptualized as “the allocation of behavior among activities” (Baum, 2010, p. 161). Put more simply, choice is engaging in a selection response when multiple options are concurrently available. Choice is frequently embedded in the practice of applied behavior analysis by arranging opportunities for clients to select the activities and stimuli to which they will be exposed and with which they will engage. Sometimes choice in clinical practice takes the form of an antecedent (i.e., proactive) event, such as allowing the client to choose which work tasks they want to engage in first. At other times, choice in clinical practice may take the form of a consequence (i.e., reactive) event, such as arranging for a client to earn access to choosing which activities they will engage in during their break after completing work.

Facilitating opportunities for clients to make choices in relation to their treatment is important from an ethical and treatment effectiveness perspective. Acknowledging and supporting client choice is explicitly listed as a critical component of treating clients with compassion, dignity, and respect within the Ethics Code for Behavior Analysts (Behavior Analyst Certification Board, 2020; Peterson et al., 2021). Choice is important from a compassion, dignity, and respect standpoint because it is central to involving clients in therapeutic decisions and helping them gain as much independence and autonomy as possible (Morris et al., 2024; Rajaraman et al., 2023). Thus, important clinical and research practices like obtaining assent and consent from clients are predicated on the client having the skills, opportunity, and support needed to engage meaningfully in choices pertaining to their treatment.

Several research findings also suggest that humans prefer choice opportunities over no‐choice opportunities (Fisher et al., 1997; Tiger et al., 2006) and that when choice opportunities are integrated into treatment, client outcomes improve (Cannella et al., 2005; Kestner et al., 2023). For example, increasing choice opportunities has been shown to increase functional skills, such as on‐task behavior (Dunlap et al., 1994; Watanabe & Sturmey, 2003) and assignment completion (Stenhoff et al., 2008), and to decrease challenging behavior (Dunlap et al., 1994; Lory et al., 2020; Rispoli et al., 2013). Thus, behavior analysts should attempt to embed choice in treatment as much as possible.

Although choice is important in clinical contexts, successfully arranging choice can be complicated because it is notably complex. Seemingly small differences in choice arrangements, like the number of options and the delay to choice outcomes, have the potential to affect the efficacy of choice as a component of treatment and clients' preferences for choice. Consequently, any differences in the way researchers and practitioners provide choice opportunities have the potential to lead to different outcomes. Thus, understanding the parameters of choice arrangements is essential to their successful use.

Several reviews of choice as an intervention have been conducted to identify important parameters of choice in practice. These reviews have evaluated the effects of choice in daily living situations (Kestner et al., 2023; Lancioni et al., 1996); choice across vocational and domestic activities, choice of academic activities, and choice of leisure, recreational, and social activities (Kern et al., 1998); the influence of choice on challenging behavior (Cannella et al., 2005); choice in relation to preference assessments (Tullis et al., 2011); and choice as differential reinforcement (Kestner et al., 2023). However, previous reviews provided limited information about the influence of choice parameters on preference for choice. Therefore, the primary purpose of this study was to review behavior‐analytic articles that evaluated preference for choice by incorporating both choice and no‐choice conditions into their study. The secondary purpose was to provide future directions for practice and research based on these findings, with a focus on providing practical considerations for those interested in embedding choice into treatment. To meet the objectives, this review was used to address the following two research questions. First, what are the experimental arrangements of choice and no‐choice conditions in the behavior‐analytic literature? Second, what factors influence the preference for choice or no choice?

## REVIEW OF CHOICE AND NO‐CHOICE ARRANGEMENTS

**FIGURE 1:**
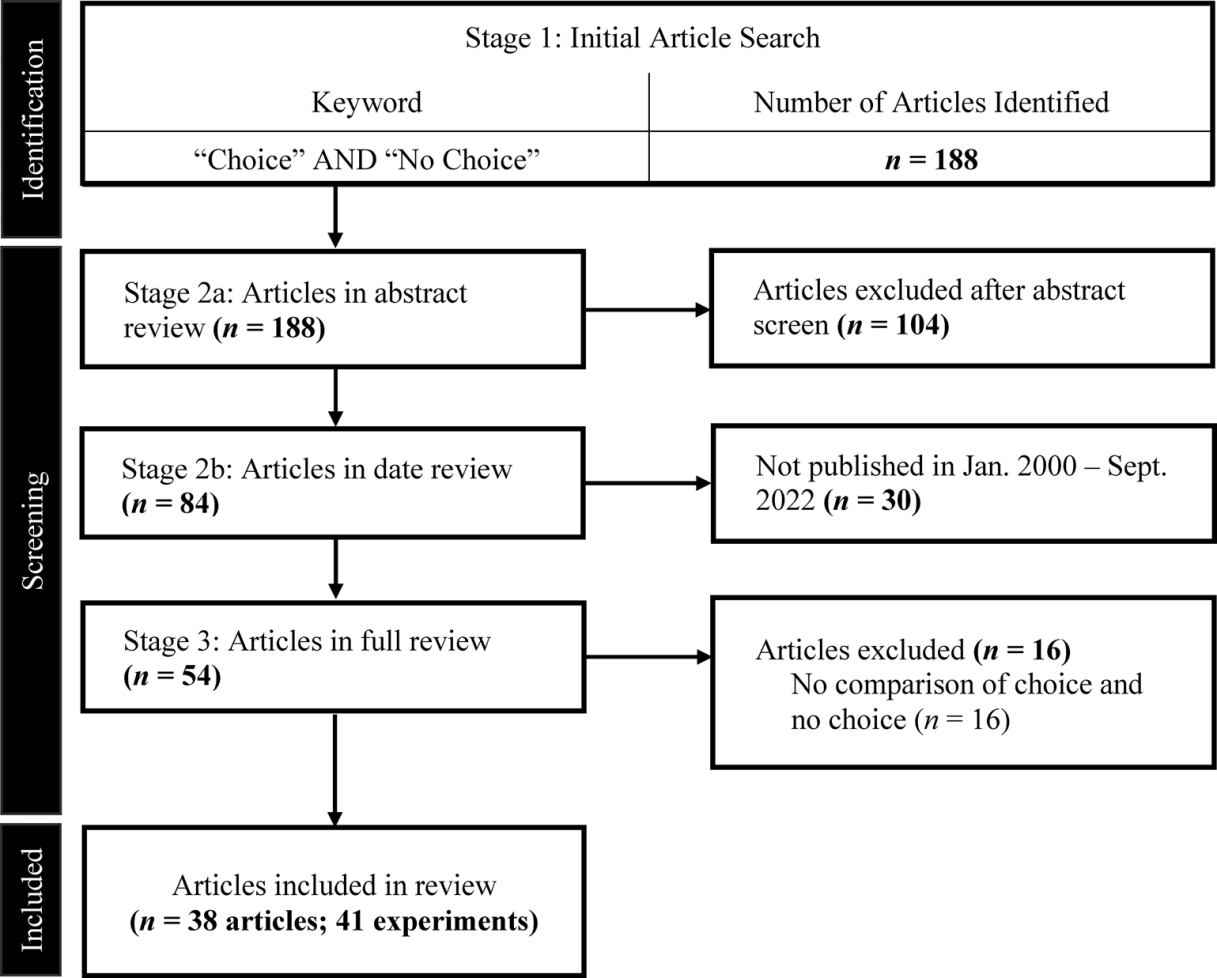
Schematic overview of the data search and data analysis process. ABAI = Association for Behavior Analysis International. BACB = Behavior Analyst Certification Board.

We reviewed behavior‐analytic literature to identify articles that compared choice and no‐choice conditions. Journals listed on the Association for Behavior Analysis International and Behavior Analyst Certification Board websites were searched using the search terms “choice” and “no choice.” Table 1 includes a list of the journals included in the review. The procedure mimicked previous studies that have searched articles in behavior‐analytic journals (Morris et al., 2021). Choice is a widely studied topic across disciplines such as psychology, economics, and political science, to name a few. Although there is value in understanding how other disciplines discuss choice (e.g., Castillo, Sun, Frank‐Crawford, & Borrero, 2022; Castillo, Sun, Frank‐Crawford, Rooker, et al., 2022; Loewenstein & Prelec, 1991), our procedures were designed to narrow our search to behavior‐analytic journals to focus on a behavior‐analytic interpretation of choice and to use the findings to better inform practice within the field. During the initial search, we identified articles that included the terms “choice” and “no choice” in any part of the article. Following the initial search, the title and abstract of each article were reviewed for the following inclusion criteria: the study or studies (a) compared choice and no‐choice conditions (i.e., there was active manipulation of choice and no‐choice conditions), (b) included human participants of any age, and (c) was written in English. Next, the publication dates were reviewed to include only articles that were published between January 2000 and September 2022. The articles included in the full review were limited to those that were published between January 2000 and September 2022 because we wanted our review to represent the latest practices involving choice. Articles that were not excluded via the title, abstract, and date review were then fully reviewed for the same inclusion criteria. Figure 1 summarizes the stages of the search and the number of articles identified and included in each stage.

**TABLE 1:** Publishing journals.

Journal	Number of articles (Experiments)
*Behavior Analysis in Practice*	3
*Behavior Modification*	1
*Behavioral Interventions*	3
*Education and Treatment of Children*	3
*European Journal of Behavior Analysis*	1
*Journal of Applied Behavior Analysis*	16 (19)
*Journal of Behavioral Education*	1
*Journal of the Experimental Analysis of Behavior*	3
*Journal of Positive Behavior Interventions*	4
*The Analysis of Verbal Behavior*	1
*The Psychological Record*	2

*Note*: The number of experiments was only denoted if the number of experiments was different from the number of articles for the respective journal.

Articles that met the inclusion criteria were analyzed and coded. Each experiment included in the articles was analyzed and coded for the general characteristics of the literature, the experimental arrangement that was used to evaluate the differential effects of choice and no choice (Research Question 1), and the preference assessments used to evaluate preference for choice and no‐choice conditions (Research Question 2). Tables 2, 3, 4 include the definitions of each component coded for the general characteristics of the literature, experimental arrangement for choice and no choice, and preference for choice, respectively.

**TABLE 2:** Definitions of article analysis components for general characteristics of the literature.

Component	Definition
Journal name	The name of the journal.
Publication year	The year the article was published online or in print depending on the current status of the manuscript.
Demographics (race and ethnicity)	Information on the participants' race and ethnicity (e.g., White, Black, Hispanic, Asian, multiracial, or information unavailable).
Setting	The experimental context in which the experiment was conducted (e.g., school, university, clinic home, or other).
Study type	Although all included experiments were comparative, they were further categorized as a demonstration study, parametric analysis, or component analysis. A demonstration study is one in which researchers assessed the effects of an intervention on a target behavior. A parametric analysis is one in which the effects of different levels or dosages of an intervention on the dependent variable are assessed. A component analysis is one in which different pieces or components of an intervention package are evaluated to determine which piece affects the dependent variable.

**TABLE 3:** Definitions of article analysis components for the experimental arrangement of choice and no choice.

Component	Definition
Independent variable(s)	The primary intervention implemented to observe its effects on the dependent variable.
*Codes*	
Choice and no choice	When an experiment directly manipulated the choice and no‐choice conditions to compare the two conditions.
Differential reinforcement	When an experiment investigated the effects of differential associations with reinforcer parameters on one's preference for choice versus no choice.
Task/Activity choices	When an experiment involved an instructor providing multiple alternatives for instructional tasks or activities.
Other	Independent variable(s) that were not included in the aforementioned codes.
Dependent variable(s)	The behavior measured to observe how it changed because of the manipulation of the independent variable.
*Codes*	
Initial link selection	Selection made in the initial links of a concurrent‐chains arrangement.
completion	Engagement in a response for a predetermined period of time or completing multiple steps of instructions or tasks.
Challenging behavior	A pattern of behavior interfering with an individual's learning or engagement with others.
Academic responses	Count of discrete responses in an academic context.
On‐task behavior	Active participation in the task or activity.
Simple operant task responses	Single step, discrete responses in a nonacademic context.
Preference selection	Selection of a preferred option out of an array of alternatives in a concurrent‐operants arrangement.
Other	Dependent variable(s) that were not included in the aforementioned codes.
Teaching of prerequisite skills	Whether the researcher taught prerequisite skills needed for selection responses (e.g., attending to and reaching for the options, stimuli discrimination) prior to beginning the intervention.
Option type	The description of the type of choices provided.
*Codes for putative reinforcers as choice options*	
Edibles	Preferred food.
Tangibles	Preferred items (e.g., toys, stickers).
Activities	Preferred activity (e.g., games).
Location	Preferred place to complete a noninstructional task.
*Codes for instructional choice options*	
Academic materials	Preferred type of materials used during instruction.
Location	Preferred place to complete an instructional task.
Task sequence	Preferred order of steps to complete a task.
Antecedent instruction	Preferred instructional procedure.
Task engagement	Whether an individual engaged in a task or not.
Response topography	Preferred way to respond.
*Codes for other choice options*	
Numbers on screen	Numbers displayed on a computer screen.
Cooperate or defect	Access to a larger, delayed reinforcer (i.e., cooperate), or a smaller, immediate reinforcer (i.e., defect).
Colored cards	Colored cards displayed on a computer screen.
Array size	The number of alternatives from which to choose.
Antecedent versus consequence presentation of choice opportunity	*Antecedent* means that the experimenter provided the choice opportunity prior to each participant engaging in a target behavior. *Consequence* means that the experimenter provided the choice opportunity after the participant engaged in a target behavior.
Immediate versus delayed delivery of chosen option	*Immediate delivery* means that the experimenter delivered the chosen option immediately following a selection response. *Delayed delivery* means that the experimenter delivered the chosen option after completion of work requirement.
Baseline/Control	Some studies incorporated a separate baseline phase or control condition. In these conditions, an individual's response resulted in no reinforcement (e.g., empty plate or moving to a different activity) or neutral consequence that was not an option (e.g., praise), whereas a no‐choice condition involved a limited option (e.g., experimenter‐selected option).

*Note*: If the study specified an initial and terminal link, the terminal link was used to analyze the arrangement of the choice condition.

**TABLE 4:** Definitions of article analysis components for preference for choice.

Component	Definition
*Initial link type	The description of the type of initial link alternatives provided.
Initial link array size	The number of alternatives from which to choose in the initial link.
Number of choice preferences across participants	The number of participants preferring choice, no choice, or with no preference. No preference meant that the participant had similar preference across choice, no choice, and/or control conditions. If the purpose of the experiment was to shift preference, the initial assessment data prior to the intervention (e.g., condition with no change in reinforcer magnitude in Rost et al. [2014]) were used.
Maintenance of choice preference	When the selection of choice condition was sustained across time. This was determined by a participant successively selecting choice condition over no‐choice condition without switching preference from choice to no choice.

*Note*: *Previous studies showed that an individual's relative level of responding in the initial links reflects their preference for the contingencies in the terminal links (Catania, 1992). During the initial link, concurrently available responses associated with independent reinforcement schedules are presented. The selection of a response in the initial link determines the schedule or type of reinforcement in the terminal link.

### Intercoder agreement

Two reviewers independently conducted the initial article search, full review, and article coding. Independent reviewers completed the entire initial article search; reviewed 33.33% of articles identified through the initial search, abstract review, and date review stages for the full review; and coded 34.15% of the experiments for the coding. The first author randomly selected the articles reviewed in the full‐review and article‐coding stages. Total‐count intercoder agreement was calculated for the initial article search by dividing the smaller number of found articles by the larger number of found articles and multiplying by 100. Intercoder agreement for the initial article search was 100%. Trial‐by‐trial intercoder agreement was calculated for the full review by dividing the number of experiments with agreement by the total number of experiments and multiplying by 100. The mean agreement score for the full review was 94.44% (range: 0%–100%). Trial‐by‐trial intercoder agreement was calculated for article coding by dividing the number of components with agreement by the total number of components listed in Tables 2, 3, and 4 and multiplying by 100. The mean agreement score for the article coding was 94.83% (range: 84%–100%). Disagreements were resolved through discussions among the research team.

## RESULTS

### General characteristics of the literature

**FIGURE 2:**
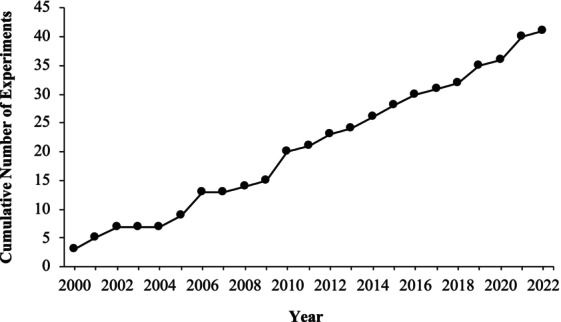
Cumulative number of experiments published between January 2000 and September 2022.

The 38 articles included in this review were published in 11 journals. The *Journal of Applied Behavior Analysis* (*n* = 19) had the highest number of experiments, followed by the *Journal of Positive Behavior Interventions* (*n* = 4). See Table 1 for the complete list of journals and the number of articles found in each. Figure 2 depicts the cumulative number of experiments included in this review. On average, 2.05 articles were published each year (range: 1–5). Out of the 322 participants across the 41 experiments, the demographic information on race and ethnicity was unavailable for 305 participants. Of the participants whose demographic information was made available, eight were White, five were Black, three were Hispanic/Latinx, and one was Asian. Experiments were conducted most frequently in a school setting (*n* = 20), followed by university (*n* = 9), clinic (*n* = 4), home (*n* = 3), residential facility (*n* = 3), and other (*n* = 3).^1^ The “other” category included online, individual cubicle, and inpatient settings. Out of the 41 experiments, two included a parametric analysis, two other experiments included a component analysis, and five other experiments were demonstration studies.

### Research Question (1): What are the experimental arrangements for choice and no choice within the behavior‐analytic literature?

The purpose of this evaluation was to identify the common strategies for experimental arrangement that were used to compare choice and no‐choice conditions. The information from this evaluation was categorized into three choice arrangements: putative^2^ reinforcers as choice options, instructional choice options, and other choice options. The definitions for each analysis are shown in Table 3, and the data are shown in Tables 5, 6, 7, respectively. An arrangement was categorized as *putative reinforcers as choice options* when the choice condition involved a preference assessment or functional analysis to determine a potential reinforcer used as the choice option. An arrangement was categorized as *instructional choice options* when the choice condition involved following an instruction or completing a task. An arrangement was categorized as *other choice options* if the choice condition did not meet the criteria for the aforementioned categories. A combined analysis across the three arrangements is provided at the end of this section.

**TABLE 5:** Putative reinforcers as choice options (*n* = 24 experiments).

References	IV	DV	Teachingprereq skills	Choice				No choice
				Option	Array Size	Ant vs. Cons	ID vs. DD	BL/Control
Ackerlund Brandt et al. – Exp 1 (2015)	Choice and no choice	Initial link selection	N	Edibles	5	A	DD	Y
Ackerlund Brandt et al. – Exp 2 (2015)	Differential reinforcement	Initial link selection	N	Edibles	2, 8	A	DD	Y
Boga & Normand – Exp 2 (2017)	Choice and no choice	Other	N	Location	4	A	ID	N
Carter (2001)	Choice and no choice	Challenging behavior, other	N	Tangibles, activities	10	A	ID	N
Drifke et al. (2019)	Differential reinforcement	Initial link selection	N	Edibles	3	A	DD	Y
Fenerty & Tiger (2010)	Choice and no choice	Initial link selection	N	Edibles	5	C	ID	Y
Geckeler et al. (2000)	Choice and no choice	Simple operant task response	N	Edibles	3	C	ID	Y
Gifford et al. (2022)	Differential reinforcement	Initial link selection	N	Edibles	3	A	DD	Y
Hanratty & Hanley (2021)	Choice and no choice	Academic response, initial link selection	N	Edibles, tangibles	21	C	ID	Y
Harding et al. (2005)	Choice and no choice	Challenging behavior, task completion: academic	N	Tangibles	2	A	ID	Y
May (2019)	Choice and no choice	On‐task behavior	N	Activities	3	A	ID	N
North & Iwata – Exp 3 (2005)	Other	Simple operant task response	N	Edibles	6	A	DD	Y
Northgrave et al. (2019)	Choice and no choice	Academic response	N	Edibles	5	C	ID	Y
Peterson et al. (2016)	Other	Academic response, initial link selection	N	Edibles	3	A, C	DD, ID	Y
Schmidt et al. (2009)	Choice and no choice	Initial link selection	N	Edibles, tangibles	5	A	DD	Y
Sellers et al. (2013)	Choice and no choice	Simple operant task response, initial link selection	Y	Edibles	4	C	ID	Y
Sran & Borrero (2010)	Choice and no choice	Academic response, initial link selection	N	Edibles	5	C	ID	Y
Tiger et al. – Exp 1 (2006)	Choice and no choice	Task completion: academic, initial link selection	N	Edibles	5	A	DD	Y
Tiger et al. – Exp 3 (2006)	Choice and no choice	Task completion: academic, initial link selection	N	Edibles	5, 10, 15	A	DD	N
Tiger et al. – Exp 4 (2006)	Choice and no choice	Task completion: academic, initial link selection	N	Edibles	8, 16	A	DD	Y
Tiger et al. (2010)	Choice and no choice	Academic response	Y	Edibles	5	C	ID	Y
Toussaint et al. (2016)	Choice and no choice	Academic response, initial link selection	N	Edibles	3	A	DD	Y
Waldron‐Soler et al. (2000)	Choice and no choice	Task completion: academic	N	Edibles, tangibles	3	C	ID	Y
Wiskow et al. (2018)	Choice and no choice	Challenging behavior, preference selection	Y	Edibles	5	C	ID	Y

*Note*: IV = independent variable; DV = dependent variable; Prereq = prerequisite; Ant (A) = antecedent; Cons (C) = consequence; ID = immediate delivery; DD = delayed delivery; BL = baseline; Y = yes; N = no, Exp = experiment.

**TABLE 6:** Instructional choice options (*n =* 14 experiments).

References	IV	DV	Teaching prereq skills	Choice				No choice
				Option	Array size	Ant vs. Cons	ID vs. DD	BL
Cole & Levinson (2002)	Choice and no choice	Other	N	Task sequence	2	A	ID	N
Daly et al. (2006)	Task/activity choice	Academic response	N	Antecedent instruction, task engagement	2, 4	A	ID	N
Deel et al. (2021)	Choice and no choice	Task completion: leisure, preference selection	N	Task sequence	5	A	ID	Y
Fenerty & Tiger (2010)	Task/activity choice	Initial link selection	N	Academic materials	5	A	ID	Y
Kern et al. (2001)	Choice and no choice	Challenging behavior; on‐task behavior	N	Task sequence	3	A	ID	N
Lory et al. (2020)	Choice and no choice	Challenging behavior	Y	Academic materials, location	Unclear	A	ID	Y
MacNaul et al. (2021)	Choice and no choice	Academic response; preference selection	N	Academic materials	2	A	ID	Y
Ramsey et al. (2010)	Choice and no choice	On‐task behavior, task completion: academic, academic response	N	Task sequence	2	A	ID	N
Rispoli et al. (2013)	Task/activity choice	Challenging behavior	N	Task sequence, location, academic materials, response topography	2, 4	A	ID	N
Romaniuk et al. (2002)	Choice and no choice	Challenging behavior	N	Academic materials	4–6	A	ID	N
Salmento & Bambara (2000)	Other	Other	N	Task sequence, task engagement	2	A	ID	N
Solis et al. (2021)	Choice and no choice	Other, preference selection	N	Academic materials	3	A	ID	Y
Stenhoff et al. (2008)	Choice and no choice	Task completion: academic; academic response	N	Academic materials	2	A	ID	N
Ulke‐Kurkcuoglu & Kircaali‐Iftar (2010)	Task/activity choice	On‐task behavior	N	Academic materials	2	A	ID	N

*Note*: IV = independent variable; DV = dependent variable; Prereq = prerequisite; Ant (A) = antecedent; Cons (C) = consequence; ID = immediate delivery; DD = delayed delivery; BL = baseline; Y = yes; N = no.

**TABLE 7:** Other choice options (*n* = 4 experiments).

References	IV	DV	Teaching prereq skills	Choice				No choice
				Option	Array Size	Ant vs. Cons	ID vs. DD	BL
Karsina et al. (2012)	Differential reinforcement	Initial link selection, other	N	Numbers on screen	8	A	ID	N
Karsina et al. (2011)	Differential reinforcement	Initial link selection, other	N	Numbers on screen	8	A	ID	N
Locey & Rachlin – Exp 1 (2012)	Other	Other	N	Cooperate or defect	2	A	ID	Y
Rost et al. (2014)	Choice and no choice	Initial link selection	Y	Colored cards	3	A	ID	N

*Note*: IV = independent variable; DV = dependent variable; Prereq = prerequisite; Ant (A) = antecedent; Cons (C) = consequence; ID = immediate delivery; DD = delayed delivery; BL = baseline; Y = yes; N = no; Exp = experiment.

#### Putative reinforcers as choice options

Table 5 displays the experimental arrangements for choice and no choice of the 24 experiments that used putative reinforcers as choice options. The independent variables included the choice and no‐choice (*n* = 19; 79.17%), differential‐reinforcement (*n* = 3; 12.50%), and other conditions (*n* = 2; 8.33%). The “other” category included posttrial and pretrial choice and a treatment package of varied reinforcers within sessions including choice of reinforcer, increased break, and intermittent reinforcement. Initial link selection was most widely used as the dependent variable (*n* = 14; 58.33%), followed by academic responses (*n* = 6; 25.00%), task completion (*n* = 5; 19.23%), challenging behavior (*n* = 3; 12.50%), simple operant task response (*n* = 3; 12.50%), on‐task behavior (*n* = 1; 4.17%), preference selection (*n* = 1; 4.17%), and other (*n* = 2; 8.33%).^3^ Dependent variables categorized as “other” included physical activity and social skills.

Three experiments (12.50%) reported the teaching of prerequisite skills prior to the experiment, such as teaching how to discriminate between different stimuli and teaching how to make selections out of concurrently available options. The most widely used choice option was edibles (*n* = 20; 83.33%), followed by tangibles (*n* = 5; 20.83%), activities (*n* = 2; 8.33%), and location (*n* = 1; 4.17%).^4^

The array size ranged from two to 21, with five being the modal array size of choice options (*n* = 9; 37.50%), followed by three (*n* = 7; 26.17%). Fifteen experiments (62.50%) provided the choice opportunity before the participant engaged in the target behavior, whereas 10 experiments (41.67%) provided the choice opportunity after the participant engaged in the target behavior. Fourteen experiments (58.33%) delivered the chosen option immediately, whereas 11 experiments (45.83%) delivered the chosen option after a delay.^5^

The no‐choice condition arrangement was examined by determining whether an experiment incorporated a separate baseline phase or control condition. Out of 24 experiments, 20 (83.33%) included a baseline/control condition. Four experiments (16.67%) included neither a baseline phase nor a control condition, meaning they directly compared choice and no choice without a neutral control (e.g., an experiment using a reversal design or an alternating‐treatment design to compare choice and no‐choice conditions without a baseline or control condition).

In general, when a putative reinforcer was used as the choice option, an experiment did not include the teaching of prerequisite skills and edibles were most widely used in an array size of five. About half of the experiments reported that the choice opportunity was provided before the participant engaged in the target behavior with immediate delivery of chosen option.

#### Instructional choice options

Table 6 displays the experimental arrangements of choice and no choice of the 14 experiments that used instructional choice options. The most used independent variables were choice and no choice (*n* = 9; 64.29%), followed by task/activity choice (*n* = 4; 28.57%) and other (*n* = 1; 7.14%). The “other” category included a staff training package. The most widely used dependent variables were academic response (*n* = 4; 28.57%) and challenging behavior (*n* = 4; 28.57%), followed by on‐task behavior (*n* = 3; 21.43%), task completion (*n* = 3; 21.43%), preference selection (*n* = 3; 21.43%), initial link selection (*n* = 1; 7.14%), and other (*n* = 3; 21.43%).^6^ The “other” category included vocational and daily living skills, choice opportunity presentation, curriculum‐based measurement, standardized reading measures, and social‐validity measures.

One experiment (7.14%) reported teaching prerequisite skills prior to the experiment. The teaching involved establishing correspondence between vocal and nonvocal behavior. The most widely used choice option was academic materials (*n* = 8; 57.14%), followed by task sequence (*n* = 6; 42.86%), task engagement (*n* = 2; 14.29%), location (*n* = 2; 14.29%), antecedent instruction (*n* = 1; 7.14%), and response topography (*n* = 1; 7.14%).^7^ The array size ranged from two to six, with two being the modal array size of choice options (*n* = 8; 57.14%), followed by four (*n* = 3; 21.43%). All 14 experiments (100%) provided the choice opportunity before the participant engaged in the target behavior and delivered the chosen option immediately. When evaluating the no‐choice condition arrangement across the 14 experiments, five experiments (35.71%) included a baseline/control condition, whereas nine experiments (64.29%) included neither.

Overall, when instructional choice options were evaluated, one experiment reported the teaching of prerequisite skills. Academic materials were most widely used in an array size of two, and all experiments reported that the choice opportunity was provided before the participant engaged in the target behavior, with immediate delivery of the chosen option.

#### Other choice options

Table 7 displays the experimental arrangements of choice and no choice of the four experiments that used choice options other than putative reinforcers and instructional choice options. This section provides preliminary information on other choice options and should be read with caution given the wide range of choice options and the small number of experiments for each choice option. The four experiments discussed in this section are qualitatively different from those under the putative reinforcer as choice options and instructional choice options categories. The experiments in this section are all basic human operant studies, whereas the other experiments are more applied. Independent variables included differential reinforcement (*n* = 2; 50.00%), choice and no choice (*n* = 1; 25.00%), and other (*n* = 1; 25.00%). The “other” category included cooperation and defect conditions.^8^ Dependent variables included initial link selection (*n* = 3; 75.00%) and other (*n* = 3; 75.00%).^9^ The “other” category included choice, points, and odds quotient^10^ and cooperation and defect selection. One experiment (25%) reported teaching prerequisite skills prior to the experiment. The most widely used choice option type was numbers on screen (*n* = 2; 50%), followed by cooperate or defect (*n* = 1; 25%) and colored cards (*n* = 1; 25%). The array size ranged from two to eight, with eight being the most widely used array size of choices (*n* = 2; 50%). All four experiments (100%) provided the choice opportunity before the participant engaged in the target behavior, and all four experiments (100%) immediately delivered the chosen option. Three experiments included neither a baseline phase nor a control condition (75%), whereas one experiment included both a baseline and a control condition (25%).

#### Combined analysis

##### Independent and dependent variables

Out of the 41 experiments, choice and no‐choice conditions were used most widely as the independent variable (*n* = 29; 70.73%), followed by differential reinforcement (*n* = 5; 12.20%). A wide range of dependent variables was used across all 41 experiments, with the initial link selection most widely used (*n* = 18; 43.90%), followed by academic responses (*n* = 10; 24.39%) and challenging behavior (*n* = 7; 17.07%).

##### Experimental arrangement

**FIGURE 3:**
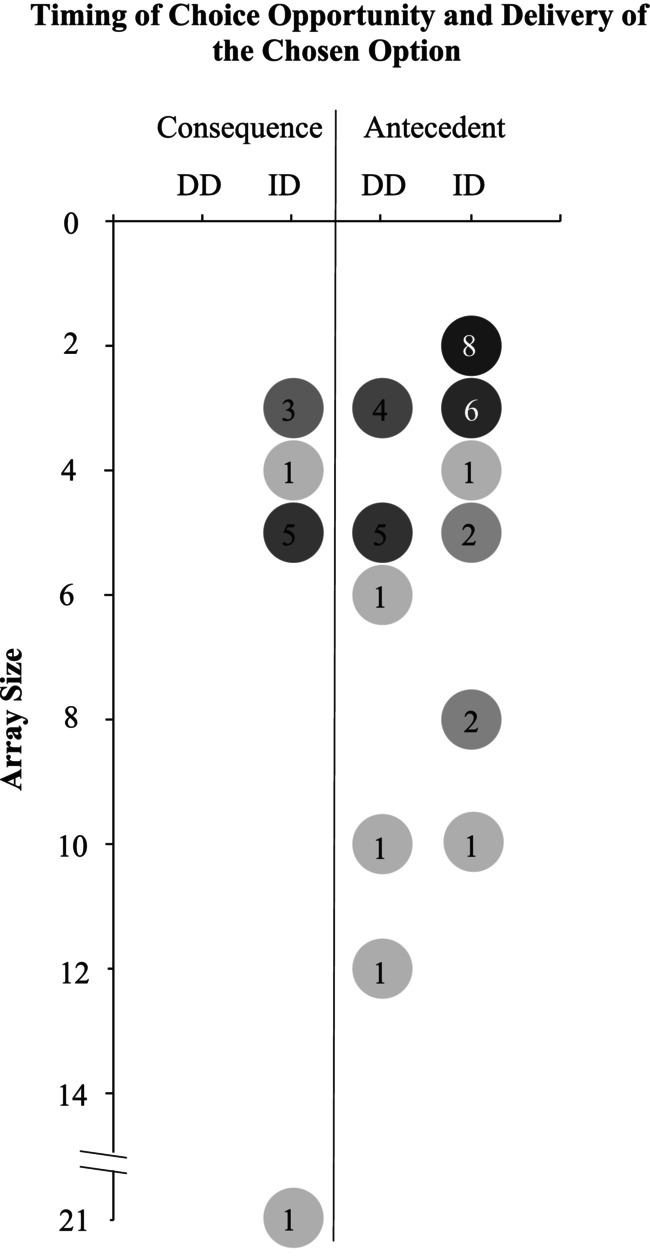
The number of experiments in each arrangement of choices. The number inside the circle indicates the number of experiments in the respective arrangement combination. Darker circles indicate multiple experiments, and lighter circles indicate fewer experiments. The total number on this figure exceeds the total number of experiments because two experiments with both immediate delivery and delayed delivery conditions were counted separately. One experiment was excluded because the array size was unclear. ID = immediate delivery; DD = delayed delivery.

Figure 3 shows a summary of the experimental arrangement of the choice conditions across 42 experiments^11^ based on the array size, antecedent or consequence presentation of the choice opportunity, and immediate or delayed delivery of the chosen option. If multiple array sizes were used in one experiment, the mean was used for this analysis. For example, Tiger et al. (2006) Experiment 3 used array sizes of 5, 10, and 15. For the analysis of the arrangements, we used 10 as the array size.

The highest number of arrangements (*n* = 8 experiments; 19.05%) included the presentation of the choice opportunity before a participant engaged in a target behavior, with an array size of two and immediate access to the selected choice option. In general, the array size was smaller when the choice opportunity was provided before a participant engaged in a target behavior than when the choice opportunity was provided after a participant engaged in a target behavior. No experiments provided the choice opportunity after a participant engaged in a target behavior along with delayed access to the selected choice option.

### Research Question (2): What factors influence the preference for choice or no choice?

Table 8 displays the data from 22 experiments (54.66% of all experiments) that evaluated the participants' preference for the choice versus no‐choice condition (see Table 4 for a review of the definitions used in the analysis). Out of 22 experiments, 16 experiments (72.73%) used putative reinforcers as choice options, three experiments (13.64%) used instructional choice options, and three experiments (13.64%) used other choice options. Initial links were used to evaluate preference in 18 out of 22 experiments (81.82%). Four experiments (18.18%) used other arrangements including a concurrent‐operants arrangement (Deel et al., 2021; MacNaul et al., 2021), verbally asking about the participant's preference (Wiskow et al., 2018), or no report of how preference was measured (Solis et al., 2021). All 18 experiments (100%) that used an initial link to evaluate preference included a selection response to evaluate the initial link response. Fifteen (83.33%) involved a touching response of a physical stimulus (e.g., worksheet), and three experiments (16.67%) involved a clicking response using a computer mouse. The array size ranged from two to four with a mode of three (*n* = 13; 68.42%).

**TABLE 8:** Preference assessments (*n =* 22 experiments).

References	Choice category	Initial link		Number of participants preferring choice	Number of participants preferring no choice	Number of participants with no preference	Maintenance of preference
		Type	Array size				
Ackerlund Brandt et al. – Exp 1 (2015)	R	Flashcard selection	3	20 out of 30	0 out of 30	10 out of 30	N
Ackerlund Brandt et al. – Exp 2 (2015)	R	Flashcard selection	3	8 out of 11	0 out of 11	3 out of 11	*N
Deel et al. (2021)	R	‐	‐	2 out of 3	1 out of 3	0 out of 3	N
Drifke et al. (2019)	R	Worksheet selection	3	5 out of 5	0 out of 5	0 out of 5	*N
Fenerty & Tiger (2010)	R	Index card selection	4	3 out of 4	0 out of 4	1 out of 4	N
Fenerty & Tiger (2010)	I	Index card selection	4	2 out of 4	0 out of 4	2 out of 4	N
Gifford et al. (2022)	R	Worksheet selection	3	1 out of 2	1 out of 2	0 out of 2	*N
Hanratty & Hanley (2021)	R	Wingding symbol selection	3	5 out of 6	0 out of 6	1 out of 6	N
Karsina et al. (2011)	O	Message box selection	2	7 out of 7	0 out of 7	0 out of 7	*N
Karsina et al. (2012)	O	Message box selection	2	4 out of 6	2 out of 6	0 out of 6	*N
MacNaul et al. (2021)	I	‐	‐	‐	‐	‐	‐
Peterson et al. (2016)	R	Card selection	2	4 out of 4	0 out of 4	0 out of 4	Y
Rost et al. (2014)	O	Card selection	2	12 out of 14	1 out of 14	1 out of 14	‐
Schmidt et al. (2009)	R	Worksheet selection	3	7 out of 8	0 out of 8	1 out of 8	N
Sellers et al. (2013)	R	Task materials selection	3	2 out of 4	2 out of 4	0 out of 4	N
Solis et al. (2021)	I	‐	‐	4 out of 4	0 out of 4	0 out of 4	‐
Sran & Borrero (2010)	R	Worksheet selection	3	3 out of 3	0 out of 3	0 out of 3	‐
Tiger et al. – Exp 1 (2006)	R	Worksheet selection	3	5 out of 6	1 out of 6	0 out of 6	N
Tiger et al. – Exp 3 (2006)	R	Worksheet selection	3	2 out of 3	0 out of 3	1 out of 3	N
Tiger et al. – Exp 4 (2006)	R	Worksheet selection	3	3 out of 3	0 out of 3	0 out of 3	**N
Toussaint et al. (2016)	R	Card selection	3	3 out of 3	0 out of 3	0 out of 3	Y
Wiskow et al. (2018)	R	‐	‐	1 out of 1	0 out of 1	0 out of 1	Y

*Note*: A hyphen (−) indicates that the respective information was unavailable. An asterisk (*) indicates when differential reinforcement was used as an intervention and successfully switched preference. Two asterisks (**) indicate when increased effort required to earn a reinforcer switched preference. R = putative reinforcers as choice options; O = other choice options; I = instructional choice options; Y = yes; N = no; Exp = experiment.

Out of 131 participants across the 22 experiments, 103 participants (78.63%) preferred choice, eight participants (6.11%) preferred no choice, and 20 participants (15.27%) showed no preference. Out of the 22 experiments, 18 (81.82%) were evaluated for maintenance of preference for choice across time (four studies were excluded because preference was not reported across sessions or days but at an aggregate level). Fourteen out of the 18 experiments (77.78%) reported that the preference was not maintained across time and that there was a shift in preference. The shift in preference was demonstrated when differential reinforcement history successfully switched an individual's preference (Ackerlund Brandt et al., 2015; Drifke et al., 2019; Gifford et al., 2022; Karsina et al., 2011, 2012) or increased effort switched an individual's preference (Experiment 3 of Tiger et al., 2006). Differential reinforcement was evaluated through multiple parameters of reinforcement including preference for reinforcers (e.g., high‐ versus low‐preference items; Ackerlund Brandt et al., 2015; Drifke et al., 2019), reinforcer magnitude (Drifke et al., 2019), immediacy of reinforcer delivery (Gifford et al., 2022), and odds of winning a game (Karsina et al., 2011, 2012).

## DISCUSSION

The main purpose of this research was to review and summarize the arrangements of choice within experimental evaluations of choice and no choice in articles published in behavior‐analytic journals. Although several previous reviews evaluated important components of choice procedures, this review was the first to provide information about the specific arrangement for evaluating choice in comparison with no choice. We provided a summary of the general characteristics of the literature and discussed the two main findings, implications for practice and research, and limitations of the current review.

The overall rate of experiments published was steady across the years of this review, with 21 experiments published during the first 11 years and 20 experiments during the last 11 years covered. Aspects of the general characteristics of the literature were consistent with those of other behavior‐analytic research, such as underreporting participants' race and ethnicity (see Jones et al., 2020). The omission of race and ethnicity data limits the overall generality of the information reported in this review because it is unclear whether the procedures used in the reviewed studies were successful across these demographic variables. We strongly encourage future researchers to report the demographic variables, especially race, ethnicity, and income level, when reporting participant information. Studies should also report the type and level of developmental disability given that choice is often arranged in programming for those with developmental disabilities. Additional information would allow one to analyze the differential effects of choice across demographics beyond gender and age and to develop a culturally sensitive, individualized choice arrangement.

The review of the experimental arrangement and preference‐assessment strategies described in published studies comparing choice and no‐choice conditions resulted in several interesting findings. Based on our analyses, the current state of choice arrangements informs us of two main findings discussed below.

### A wide variety of choice arrangements

Although choice is ubiquitous in many ways, our analyses show that the variables that affect choice are manifold and garner many unanswered questions. For example, a sizable difference in the mean choice array size was found between studies that evaluated choice as a putative reinforcer and those that evaluated choice as a component of instruction. When the experimental arrangements were divided into three different option type categories (i.e., putative reinforcers as choice options, instructional choice options, and other choice options), the differences between the choice arrangements became more pronounced. The evaluation of choice as a putative reinforcer primarily involved edibles and tangibles as choice options within a larger array of 2 to 21 (mode: 5). On the other hand, the evaluation of choice as a component of instruction primarily used academic materials and task sequences as choice options within a smaller array of two to six (mode: 2). Although the difference in choice options was to be expected, the differences in the array sizes are noteworthy because such data may imply that the type of choice option is an important variable to consider when determining array sizes.

Previous findings have shown that array size may affect the value of reinforcers. For example, larger array sizes have been shown to diminish the reinforcing value of reinforcers for neurotypical preschoolers (Miller et al., 2017) and to diminish the reinforcing value of choice for adults (Reed et al., 2011). However, these findings should be interpreted cautiously when working with individuals with developmental disabilities, as studies involving individuals with autism spectrum disorder showed a greater preference for a larger array size (Fernandez et al., 2022; Tiger et al., 2006). One consideration is that choice used as a component of instruction may be more sensitive to the paradox of choice (Schwartz, 2004)^12^ than choice used as a putative reinforcer because there are more potentially aversive variables involved with choosing work tasks than putative reinforcers.

Another interesting variation related to the experimental arrangement for choice was when the choice opportunity was provided. Studies evaluating choice as a component of instruction uniformly presented the choice opportunity before the participant engaged in a target behavior. This was not the case when choice was evaluated as a putative reinforcer, as 62.50% of the experiments presented the choice opportunity before the participant engaged in the target behavior and 41.67% presented it after.^13^ Similarly, the timing of the delivery of the chosen option varied. All experiments evaluating choice as a component of instruction involved immediate delivery of the chosen option. However, when evaluating choice as a putative reinforcer, 58.33% of the experiments immediately delivered the chosen option, whereas 45.83% provided it after a delay.

Typically, choice opportunities evaluated as part of instruction are presented as an antecedent. For example, providing two or more variations of a task (e.g., worksheet with numbers or shapes) prior to the beginning of a session. The chosen option is also expected to be delivered immediately, as a person cannot begin the task until they have the task. However, the variation across choice as putative reinforcers warrants further investigation. To date, there is limited experimental literature directly comparing the effects of the timing of the presentation of choice opportunity and the delivery of the chosen option on the terminal link performance or preference. When provided with a choice of two concurrent options (e.g., Would you like to play outside or read a book?), people tend to select the more preferred stimulus (DeLeon & Iwata, 1996). However, studies have also shown that when choice is part of a sequence of outcomes (e.g., Which would you like to do first: play outside or read a book?), people prefer an improving series of consequences, a phenomenon known as negative time preference (Castillo, Frank‐Crawford, Liesfeld, et al., 2022; Castillo, Sun, Frank‐Crawford, & Borrero, 2022). It is possible that the difference in how choice is framed may affect responding, but more research is needed. Other studies have also shown that individuals tend to devalue delayed reinforcers, or said differently, prefer immediate reinforcers when choice involves two concurrent options (Rachlin et al., 1991), and individuals prefer to accumulate reinforcers or, in other words, produce larger, continuous access to reinforcers delivered later (Frank‐Crawford et al., 2019). Taken together, the timing of the delivery of the choice option may affect an individual's preference for choice. Thus, finding the optimal timing may be crucial to increasing the preference for and effectiveness of choice and there should be further research in this area.

There may be several reasons for variations in choice arrangements. First, behavior analysis as a field focuses on tailoring assessment and treatment to individual needs (Baer et al., 1968). Thus, choice arrangements in behavior‐analytic articles may be individualized for every participant. Idiosyncratic choice arrangements potentially increase treatment efficacy and address potential ethical considerations that may come with different settings and available resources. For example, in terms of available resources, a practitioner may provide two options of reinforcers in classroom settings with limited resources such as time and money, whereas the array size may be larger when a practitioner works in a more resource‐abundant setting. In addition, a client may only prefer a limited number of activities, whereas another client may have a plethora of preferred activities. The former client may only need an array size of three, for example, whereas the latter client may benefit from having a larger array size.

A second reason for the variations in choice arrangements is that some of the studies may have been exploratory or preliminary. These experimenters may have used arbitrarily selected numbers or arrangements to assess the feasibility of an assessment procedure or the implementation of novel interventions (Leon et al., 2011). Exploratory and preliminary studies are valuable in informing future research and practice, and, understandably, studies may do this initially before conducting larger‐scale studies. Some experiments included in the current review disclosed the preliminary nature of the exploration (Lory et al., 2020; Wiskow et al., 2018). However, given that this was outside the scope of the purpose, we did not differentiate the types of studies.

The last reason for the variations in choice arrangements may be simply the lack of guidance on how to arrange choice best tailored for an individual. It is important that behavior analysts integrate the best available empirical data with their clinical expertise and client values and context (Slocum et al., 2014). As discussed above, variations without empirical support may compromise the efficacy of the choice arrangements in treatment and make replications across studies difficult. To this end, we will discuss developing systematic guidelines for arranging choice conditions below (Implications for practice and research).

### Learning history and response effort affect preference for choice

The evaluation of preference for choice provided insight into the variables affecting whether people prefer having choice, prefer having no choice, or have no particular preference. Within the preference assessment component of this review, an overwhelming majority of participants showed a preference for choice (103 out of 131 participants). This finding is in line with previous literature indicating that humans prefer choice opportunities over no‐choice opportunities (Fisher et al., 1997; Tiger et al., 2006) and supports various organizations' advocacy for integrating choice into support planning for individuals with developmental disabilities with the goal of emphasizing compassion, dignity, and respect (Behavior Analyst Certification Board, 2020; The Arc, n.d.). An interesting finding was that there was still a small number of participants who prefered no choice (eight out of 131 participants) or who showed no particular preference across the different choice conditions (20 out of 131 participants). Furthermore, a majority of the studies showed that there was a lack of maintenance in the participants' preference for choice.

Individual differences stemming from different learning histories may explain the differential preferences for choice. For example, several studies in this review demonstrated the effects of differential reinforcement history on preference for choice and no choice (Ackerlund Brandt et al., 2015, Experiment 2; Drifke et al., 2019; Gifford et al., 2022; Karsina et al., 2011, 2012). The successful shift in preference highlights how malleable preference for choice and no choice can be and that the arrangement of the environment along with one's reinforcement history of choice is critical in explaining one's preference. Other aspects of learning history may also affect preferences for choices but may be more difficult to identify, like temporally distant events. More research is needed on the influence of verifiable learning histories, like recent reinforcement histories, and ambiguous learning histories, like temporally distant events, on the preference for choice and no choice.

The response effort required to contact a reinforcer may also explain the inconsistent preference for choice. The findings in this review suggested that if the response effort for the choice condition increases substantially, an individual may prefer the no‐choice condition that requires less response effort. For example, Experiment 4 of Tiger et al. (2006) showed that all three participants shifted their preference from choice to no choice when the work requirement in the terminal link of the choice condition increased. This is consistent with findings from several other studies evaluating children's preference for contingent reinforcement and noncontingent reinforcement (Luczynski & Hanley, 2009, 2010). Although children generally prefer contingent reinforcement over noncontingent reinforcement under a continuous schedule of reinforcement, a shift in preference from contingent reinforcement to noncontingent reinforcement was observed when the schedule of reinforcement was progressively thinned.

Choice, or being able to express one's preference, is critical to developing an effective behavior support plan and a meaningful way for clients to be involved in their own treatment decision‐making process (Rajaraman et al., 2023). Thus, once we identify the contingencies affecting one's choice preference, we can more effectively tailor the choice condition to that individual to promote client involvement and treatment effectiveness.

### Implications for practice and research

Our findings provide directions for future practice and research. First, when arranging choice conditions, we encourage practitioners to determine the option type. This review found different arrangements of choice based on the option type. For example, array sizes of choice varied more when choice was evaluated as a putative reinforcer than when choice was evaluated as a component in instruction. Thus, practitioners should first determine the option type they will use in the choice arrangement. Then, they should arrange the other variables such as array size, the timing of choice opportunity, and delivery of the chosen option as laid out in the following paragraphs.

Second, when considering the array size, we encourage practitioners to begin with a smaller array size and increase it as needed. As mentioned above, a larger array size could diminish the reinforcing value of each option (e.g., Miller et al., 2017), so it may be beneficial to begin with a smaller array size. Our review showed that the modal array size was five when choice was evaluated as a putative reinforcer and two when choice was evaluated as a component in instruction. Unfortunately, as far as the authors know, there are no guidelines on the recommended array size for setting up the choice condition initially. We preliminarily suggest that practitioners start with an array size of five or smaller for a putative reinforcer and two for instructional choice options. There are also studies that have shown that a larger array size increases the value of choice to a greater degree than a smaller array size (e.g., Tiger et al., 2006). That is, individuals preferred having more options to choose from rather than fewer. If there is limited client responding when provided with a smaller array size, a practitioner should incrementally increase the array size to promote choice. This is a general suggestion based on an aggregate result. We strongly recommend that practitioners consider the client's individual characteristics and responding when deciding and adjusting the array size. To better understand the effects of the different array sizes in choice, more parametric studies should be conducted within and across different option types.

Third, when considering the timing of choice opportunity, our findings suggest that there is a potential advantage to providing putative reinforcers as choice options before the client engages in a target behavior. Peterson et al. (2016) showed that three out of four participants preferred choice opportunities provided before engaging in the target behavior and two out of four participants performed at a higher rate when choice opportunities were provided before engaging in the target behavior. Despite the potential advantage, a practitioner may want to provide the choice opportunity after the target response (e.g., task completion) if they wish to identify the most highly preferred reinforcer at the time of selection.

Also, it may be beneficial to provide choice opportunities involving putative reinforcers over instructional choice options, if possible. Fenerty and Tiger (2010) showed that individuals prefer the choice of consequence (i.e., putative reinforcer as choice option) over the choice of task (i.e., instructional choice option). However, it is important to note that this suggestion is based on preferences rather than some measure of performance (e.g., response rate). Information on preference allows practitioners to understand what a client “likes” but may not necessarily inform practitioners of how a client will perform given the preferred condition. It is possible that the most preferred condition results in low levels of behavior. Supplementing information on preference with information on performance will provide a richer source of information that allows practitioners to assess whether preferred conditions result in differentially optimal performance. Previous findings support this point, as they showed that choices and response rates were controlled by different dimensions of the reinforcer and there may be an aspect of performance not revealed when evaluating only preferences (Neuringer, 1967). When evaluating preference, future research should incorporate a measure that captures the performance of the socially significant target behavior and systematically compare the effects of different timing of choice opportunities on the performance.

Additionally, when considering the timing of the delivery of the chosen option, delay should be minimal. Studies have shown that increases in the delay of reinforcer delivery can lead to decreased reinforcer consumption (i.e., fewer reinforcers obtained), similar to when work requirement is increased (Bauman, 1991; Hursh et al., 2013; Leon et al., 2016). Thus, if a practitioner wants to promote responding, we recommend that practitioners deliver the chosen option immediately following a selection response. There may be cases where practitioners provide the choice opportunity prior to the target response and deliver the choice after an individual emits the target response. In these cases, some delay is acceptable. Leon et al. (2016) showed that delayed food delivery (i.e., up to 60 s) produced responding that was similar to that with immediate food reinforcement. Similarly, Sy and Vollmer (2012) showed that delays up to 40 s produced an acquisition rate similar to that when food and toys were delivered immediately. Nevertheless, there is a need for more studies evaluating the optimal timing of the delivery of the chosen option. As a starting point, future researchers should evaluate the effects of immediate versus delayed delivery of the chosen option across the different choice options on an individual's response rate and preference.

Overall, the variations in the arrangements found in this study indicate a need for systematic guidelines for increasing preference for choice with the goal of increasing treatment efficacy. The goal of the guidelines is not simply to maximize choice opportunities but to increase the efficacy of treatment while ensuring client care with compassion, dignity, and respect through strategic arrangement of choice based on individual characteristics. Developing clear guidelines when enough research is available may serve as a catalyst for practitioners to arrange choice conditions most effectively and ethically and to ensure the replicability of effective procedures.

This review also evaluated preference for choices and found inconsistency in preference for choice over no choice. Based on our findings, we strongly encourage practitioners to consider the individual learning histories and the response effort associated with choice when arranging the choice condition. Before doing so, practitioners should consider whether a client has the prerequisite skill to engage in choice. Our review identified only a handful of studies reporting prerequisite skills instruction prior to allowing a participant to engage in choice opportunities. Without ensuring that a client has a clear understanding of the contingencies, some clients may struggle to make selections or select an option that may not reflect their preferences. Thus, it may be necessary for a practitioner to ensure that a client can differentiate the different conditions, engage in a selection response, and understand the consequences associated with the different conditions. If a prerequisite skill for choice is absent, we encourage a practitioner not only to expose them to the conditions but also to directly teach the skills prior to arranging a choice condition.

Once the prerequisite skills are addressed, practitioners should start to evaluate the individual learning histories and the response effort associated with choice. We encourage practitioners first to analyze the immediate reinforcement history. As mentioned above, considering known variables (e.g., reinforcement history) and ambiguous variables (e.g., temporarily distant events) would help incorporate a client's preference into the treatment context. Like providing choice, incorporating preferences would be another way to ensure compassion, dignity, and respect in relation to treatment.

Additionally, a majority of the assessments evaluating the preference for choices were incorporated into studies evaluating the choice as a putative reinforcer rather than as a component of instruction or some other arrangement. The findings suggest that preference is seldom evaluated in studies that evaluate choice as a component of instruction. Considering the overall positive influence that choice seemed to have on instruction, future research should evaluate the preference for choices when choice is a component of instruction and investigate the effects of choice on academic performance (e.g., accuracy on math problems) and performance on academic‐related behaviors (e.g., on‐task behavior, task completion).

### Limitations

One limitation was that we only searched the behavior‐analytic journals listed on the Association of Behavior Analysis International and Behavior Analyst Certification Board websites. Due to the narrow search, there may have been behavior‐analytic articles or articles relevant to the research question not included. Given the broad use of choice arrangements in different settings, there may have been articles in other education or clinical journals. Future researchers should broaden the search using databases (e.g., PsycInfo and ERIC) and conduct a systematic review to identify studies that may provide additional information.

Furthermore, the search terms used could have resulted in studies involving no‐choice conditions not being captured in this review. Studies may have used baseline or control conditions to reflect a no‐choice condition without using the term that we searched for—“no‐choice”—to describe the condition. These studies that did not directly use the search term “no choice” would not have been included in the initial article search. Therefore, future studies should expand the search by using more inclusive search terms.

### Conclusions

We hope that after more research is conducted on choice, general guidelines for embedding choice into practice can be created. Guidelines and best practices on choice have the potential to affect several aspects of practice including reinforcer identification, instruction delivery, client involvement in therapeutic decisions, treatment selection, and other features of ethical and sound intervention. Thus, research on choice is important to and complimentary of other growing areas of research like assent with individuals with communication difficulties (Morris et al., 2021), differential reinforcement of alternative behavior without extinction (Briggs et al., 2019), and resurgence (Shahan & Craig, 2017).

Behavior analysts should continue to arrange choice opportunities for their clients as much as possible, with careful consideration of what types of choice and arrangement of choice options will be beneficial for their clients. The results of this review, combined with the previous literature reviews on the topic of choice, provide a foundational basis that behavior analysts should use as a starting point when making individualized decisions related to embedding choice in practice.

## CONFLICT OF INTEREST STATEMENT

The authors declare no conflict of interest.

## ETHICS APPROVAL

No human or animal subjects were used to produce this article.

## Data Availability

Data reviewed in the current article are available from the corresponding author upon reasonable request.

## Footnotes

^1^
The number of experiments when evaluating settings exceeded the number of experiments included in the current review because Rispoli et al. (2013) conducted their study in both a home and school setting.

^2^
We use the modifier “putative” in front of “reinforcer” throughout the article because stimuli used in choice arrays were often assumed to increase the likelihood of a specific behavior but the effects were not always empirically demonstrated.

^3^
The total number of experiments exceeds the total number of experiments using putative reinforcers as choice options because several experiments had multiple dependent variables. Refer to Table 5 for detailed information.

^4^
The total number of experiments exceeds the total number of experiments using putative reinforcers as choice options because four studies (Carter, 2001; Hanratty & Hanley, 2021; Schmidt et al., 2009; Waldron‐Soler et al., 2000) used multiple choice options.

^5^
The total number of experiments exceeds the total number of experiments using putative reinforcers because one study (Peterson et al., 2016) presented the choice opportunity both as an antecedent and consequence and delivered the chosen option both immediately and after a delay.

^6^
The total number of experiments exceeds the total number of experiments using instructional choice options because several experiments had multiple dependent variables. Refer to Table 6 for detailed information.

^7^
The total number of experiments exceeds the total number of experiments using instructional choice options because several experiments had multiple choice options. Refer to Table 6 for detailed information.

^8^
Locey and Rachlin (2012) used a prisoner's dilemma preparation to mimic a conflict situation where two individuals can choose to cooperate or defect. The selection of the former resulted in access to a larger, delayed reinforcer, whereas the selection of the latter resulted in a smaller, immediate reinforcer.

^9^
The total number of experiments exceeds the total number of experiments using putative reinforcers because two studies (Karsina et al., 2011, 2012) included multiple dependent variables.

^10^
Karsina et al. (2011, 2012) defined choice quotient as the initial link selection during choice trials, points quotient as which terminal link was followed by more points during all trials, and odds quotient as the terminal link that corresponded with better odds of point delivery in a computer‐based game that used a concurrent‐chains arrangement.

^11^
The total number of experiments analyzed in this section exceeds the total number of experiments included because two experiments (Fenerty & Tiger, 2010; Peterson et al., 2016) with both immediate and delayed delivery of chosen option conditions were counted separately and one experiment (Lory et al., 2020) was excluded because the array size was unclear.

^12^
The paradox of choice is the concept that as number of options within a choice increases, the probability of an organism engaging in the choice decreases (Schwartz, 2004).

^13^
The total number of experiments exceeds the total number of experiments using putative reinforcers because one study (Peterson et al., 2016) presented the choice opportunity as both an antecedent and consequence.
